# Investigation of In Vitro Endocrine Activities of *Microcystis* and *Planktothrix* Cyanobacterial Strains

**DOI:** 10.3390/toxins12040228

**Published:** 2020-04-04

**Authors:** Vittoria Mallia, Lada Ivanova, Gunnar S. Eriksen, Emma Harper, Lisa Connolly, Silvio Uhlig

**Affiliations:** 1 Toxinology Research Group, Norwegian Veterinary Institute, Ullevålsveien 68, N-0454 Oslo, Norway; lada.ivanova@vetinst.no (L.I.); gunnar.eriksen@vetinst.no (G.S.E.); silvio.uhlig@vetinst.no (S.U.); 2 Department of Chemistry, University of Oslo, P.O. Box 1033, N-0315 Oslo, Norway; 3 Institute for Global Food Security, School of Biological Sciences, Queen’s University Belfast, Belfast BT9 5DL, UK; eharper02@qub.ac.uk (E.H.); l.connolly@qub.ac.uk (L.C.)

**Keywords:** cyanobacteria, endocrine disruptor, estrogenic, microcystin, reporter-gene assay, *Planktothrix*, *Microcystis*

## Abstract

Cyanobacteria are cosmopolitan photosynthetic prokaryotes that can form dense accumulations in aquatic environments. They are able to produce many bioactive metabolites, some of which are potentially endocrine disrupting compounds, i.e., compounds that interfere with the hormonal systems of animals and humans. Endocrine disruptors represent potential risks to both environmental and human health, making them a global challenge. The aim of this study was to investigate the potential endocrine disrupting activities with emphasis on estrogenic effects of extracts from cultures of *Microcystis* or *Planktothrix* species. We also assessed the possible role of microcystins, some of the most studied cyanobacterial toxins, and thus included both microcystin-producing and non-producing strains. Extracts from 26 cyanobacterial cultures were initially screened in estrogen-, androgen-, and glucocorticoid-responsive reporter-gene assays (RGAs) in order to identify endocrine disruption at the level of nuclear receptor transcriptional activity. Extracts from selected strains were tested repeatedly in the estrogen-responsive RGAs, but the observed estrogen agonist and antagonist activity was minor and similar to that of the cyanobacteria growth medium control. We thus focused on another, non-receptor mediated mechanism of action, and studied the 17β-estradiol (natural estrogen hormone) biotransformation in human liver microsomes in the presence or absence of microcystin-LR (MC-LR), or an extract from the MC-LR producing *M. aeruginosa* PCC7806 strain. Our results show a modulating effect on the estradiol biotransformation. Thus, while 2-hydroxylation was significantly decreased following co-incubation of 17β-estradiol with MC-LR or *M. aeruginosa* PCC7806 extract, the relative concentration of estrone was increased.

## 1. Introduction

Cyanobacteria are cosmopolitan photosynthetic prokaryotes, also known as “blue-green algae”. In favorable conditions they can form dense blooms (water discoloration due to extended accumulation of biomass) [1], most often in freshwater, but also in brackish and marine water environments [1,2,3]. During such blooms, cyanobacteria may release a large variety of bioactive metabolites, including well-known cyanotoxins [1,4]. Therefore, they may have negative effects on water safety and quality (drinking, bathing, fishing, and recreational uses) and result in harm to invertebrates and vertebrates including humans [2,5,6,7,8,9]. Different types of cyanobacterial toxins are classified according to their toxicological target as hepatotoxins, neurotoxins, and dermatoxins. The most known and studied cyanotoxins are the microcystins (MCs), potent hepatotoxins produced by several cyanobacterial species, including *Microcystis* and *Planktothrix* spp. [10,11]. MCs are cyclic heptapeptides [12,13], which share a common core structure. Five of the seven amino acids are in general highly conserved while the other two are more variable. Thus, at least 279 different MC-congeners have been reported to date [14]. This enormous structural diversity within the class makes MCs characterization challenging, especially from a toxicological point of view.

**Figure 1:**
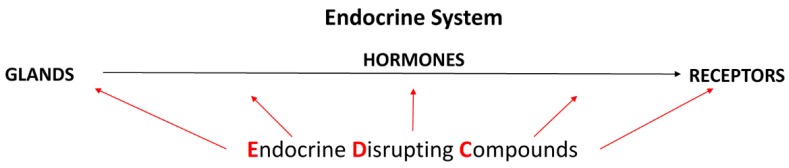
Schematic description of main components of the endocrine system. Red arrows symbolize that endocrine disruption may happen at several levels.

The so-called endocrine disrupting compounds (EDCs), or endocrine disruptors (EDs), represent another increasing environmental and health concern. EDCs are exogenous substances or mixtures named by their capability of interfering with normal endocrine (hormonal) system homeostasis. Such interferences can happen through several different mechanisms and by acting on a number of different targets of the endocrine system’s components (Figure 1), thereby causing adverse health effects in an intact organism or its progeny, as well as in entire populations [15,16].

Most studies have mainly focused on EDCs of anthropogenic origin without extensively considering those that are naturally-produced in the environment [17,18]. However, it has been reported that cyanobacterial compounds (extracts from blooms and exudates) have ED activity, mainly through activation of the estrogen receptor [19,20]. It is not entirely clear which of the different compounds present in cyanobacterial blooms exert estrogen activity. The net effect may be the result from the interaction of different cyanobacterial metabolites in the bloom, which constitute in themselves a complex mixture of known and unknown compounds, and/or other substances coexisting in the water [18,19,21].

In general, available studies on MCs activity mainly focus on just one congener, microcystin-LR (MC-LR), because of its availability, high occurrence, and high hepatotoxicity. It is, however, well known that although one or two MC-variants (not necessarily including MC-LR) are generally dominant in a single cyanobacterial strain [5,22], the majority of blooming cyanobacteria produce multiple MC-congeners [23], in addition to other bioactive compounds.

Additionally, the few studies supporting the ED potential of MCs are based on MC-LR [24,25,26]. Some other studies, with a slightly broader approach, have demonstrated ED activities in extracts of cultures from *Microcystis* and *Planktothrix* spp. (which are able to release MCs). However, they seem to contradict, or at least not confirm, the role of MCs in the observed ED activity as the extracts, but not pure MC-LR, had effect in the applied assays, including induction of vitellogenin in zebrafish larvae, an effect associated with exposure to compounds with estrogenic effects [27,28]. The estrogenic effects are frequently a result of activation of the estrogen receptor (agonist) or inhibition of the activation of the androgen receptor (antagonist). Reporter gene assays (RGAs) are excellent tools to measure the agonist or antagonist activity at the receptor levels. Furthermore, Stepankova and collaborators (2011) highlighted that cytotoxicity and estrogenic effects from extracts of blooming cyanobacteria containing MCs were greater than what would be expected from the extracts of pure laboratory cultures. These studies indicate that cyanobacterial compounds other than MCs are at least partly involved in the reported estrogenic effects. Furthermore, the potential mechanisms of the estrogenic effects of the cyanobacterial metabolites were likewise not studied.

MCs have previously been reported to cause reproductive toxicity [29,30,31]. Although reproduction is regulated through the endocrine system, it is debated if the reproductive effect of MC exposure is a direct effect on the endocrine system. Furthermore, there are indications that MCs interfere with the endocrine system acting on the hypothalamic–pituitary–thyroid axis in addition to the hypothalamic–pituitary–gonadal axis, which includes the estrogens (endogenous hormones related to reproduction) [32,33].

Based on the previously reported induction of vitellogenin, a clear indication of an estrogenic or anti-androgenic effect, the present study investigated potential ED effects of cyanobacterial extracts from *Microcystis* and *Planktothrix* spp. strains and pure MC-LR, with a main focus on estrogenic activity. We used cell-based models to investigate a direct effect at the receptor level (receptors are natural targets for endogenous hormones). In addition, we used a human liver microsomes (HLM) model to investigate possible interferences with cytochrome P450-mediated 17β-estradiol (herein simply referred to as estradiol) biotransformation. Estradiol is an endogenous hormone belonging to the group of estrogens (steroid hormones). Thus, this study aimed to address the following research questions and null hypotheses:

1. Do cyanobacterial metabolites interfere with hormone receptors, especially with the estrogen receptor?
    H_1-0_: Extracts from cyanobacterial cultures do not show effects on the estrogen receptor (or other hormone receptors).
2. Are there other modes of action that may result in endocrine disrupting activity of cyanobacterial metabolites, e.g., effects on cytochrome P450 mediated estradiol biotransformation?
    H_2-0_: Estradiol biotransformation is not affected by the presence of cyanobacterial culture extracts or MCs.
3. If cyanobacterial metabolites show ED activity, are MCs responsible for any of the observed effects?
    H_3-0_: MCs do not show ED activity.

The different in vitro assays were therefore chosen to take into account that there are a number of processes, receptor-mediated and non-receptor mediated, which could alter endocrine functions [34,35].

## 2. Results

### 2.1. Reporter Gene Assays (RGAs)

We started from a collection of 26 cyanobacterial strains, representative of two common cyanobacterial genera, *Microcystis* and *Planktothrix* (Table S1). They included both MC-producing and non-MC-producing strains. An initial screening was done using RGAs. These assays can measure effects on specific nuclear receptors signaling. It is a high-throughput method for analysis of steroid hormones agonists and antagonists, which are compounds able to mimic or block natural transcriptional activation of steroids by binding to their nuclear receptors. In the first part of the study we focused on effects on estrogen, androgen, and glucocorticoid receptors.

Prior to screening of the 26 cyanobacterial extracts using the RGAs, their cytotoxicity was assessed using the MTT (3-[4,5-dimethylthiazole-2-yl]-2,5-diphenyltetrazolium bromide) cell viability assay. Five assay dilutions of each sample were tested (1:400, 1:800, 1:1600, 1:3200, 1:6400) in order to establish a suitable (i.e., non-toxic) concentration range that could be used in the RGAs (data not shown). In general, there was no substantial cytotoxicity for any of the cell lines, and thus we performed the preliminary screening using the MMV-Luc estrogen-responsive cell line using the two highest concentrations (i.e., dilutions 1:400 and 1:800). However, one of the cyanobacterial extracts (NIVA-CYA 22), which showed weak cytotoxicity at the highest concentration, was therefore tested at 1:800 and 1:1600 dilution in the preliminary RGAs. The 26 cyanobacterial strains were subsequently screened using the TARM-Luc androgen-responsive, as well as TGRM-Luc glucocorticoid-responsive RGAs using the same extract aliquots and the same dilutions that were used for the estrogen-responsive RGAs.

Six strains were selected for further testing in the estrogen-responsive RGAs because they showed weak activities in the preliminary RGAs (e.g., 14–32% estrogen agonist activity relative to solvent control) (Table S2). Several of the extracts also indicated a possible androgen and glucocorticoid response, but the standard deviation of assay replicates was rather high (Tables S3 and S4). The follow-up of these preliminary results was beyond the scope of the present study and should be pursued in the future.

Another criterion for selection of the six strains to re-test in the estrogen-responsive RGAs was that they should include both MC-producers and non-producers. We aimed also to include representatives of both *Microcystis* and *Planktothrix* spp. In addition, the *M. aeruginosa* PCC7806 strain was selected for further testing because of its extensive use in research including previously reported estrogenic activity [27,36,37,38]. Thus, the seven strains included five *M. aeruginosa* strains, one *M. novacekii* strain and one *P. prolifica* strain (Table 1). Three of the strains were MC-producers, while four did not produce MCs according to literature data, and our own ELISA (Table 1) [38,39,40,41]. 

**Figure 2:**
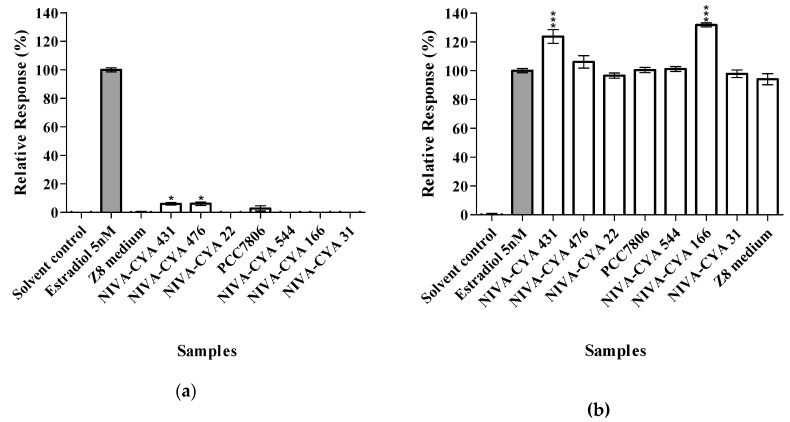
Agonistic (**a**) and antagonistic (**b**) responses of repeatedly tested cyanobacterial extracts (shown for highest concentration) and cyanobacterial growth medium (Z8) in the MMV-Luc estrogen-responsive reporter-gene assays (RGAs). Measured responses are presented relative to the cyanobacterial growth medium Z8 (**a**) and estradiol (**b**, 1.36 ng/mL or 5 nM), and expressed as the relative response ± SEM for three independent experiments (each in triplicate). One-way analysis of variance (ANOVA) and Dunnett’s multiple comparison test were used. *p* ≤ 0.05 (*), *p* ≤ 0.001 (***) vs. Z8 medium control (**a**) or positive control (**b**).

**Figure 3:**
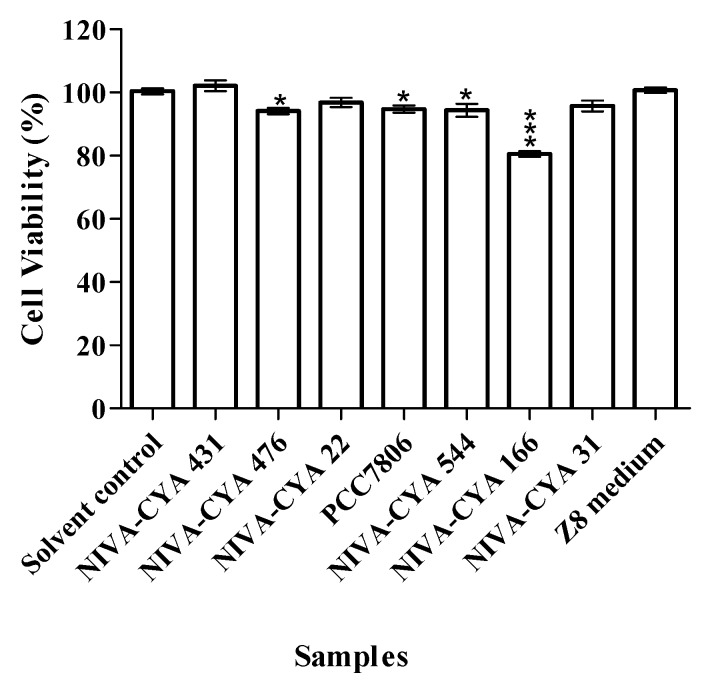
Viability of MMV-Luc cells following exposure to the seven concentrated cyanobacterial extracts and cyanobacterial growth medium (Z8) for 48 h, compared to the solvent control. Values are means of the relative viability ± SEM for three independent experiments (n = 3). One-way analysis of variance (ANOVA) and Dunnett’s multiple comparison test were used. *p* ≤ 0.05 (*), *p* ≤ 0.001 (***) vs. solvent control.

The seven extracts were reanalyzed using the estrogen-responsive RGAs in three independent experiments using new extract replicates that had been concentrated 20:1 (Figure 2). At this stage we also included cyanobacterial growth medium Z8 [42] that was extracted and concentrated using the same protocol as the cyanobacterial cultures, and the effects of the cyanobacterial cultures were expressed as relative to the Z8 growth medium control (which in itself exhibited a low estrogen agonist response). However, the estrogen agonist response for strains NIVA-CYA 431 and 476 was weak but statistically significant (P≤ 0.05) compared to that of Z8 medium (Figure 2a). Extracts from two *Microcystis* strains (NIVA-CYA 431 and 166) gave a weak but statistically significant (P≤ 0.001) enhancement of the estrogen effect from estradiol (positive control) (Figure 2b). The MTT cell viability assay was always performed in parallel to the RGAs in order to monitor potential cytotoxic effects of the cyanobacterial extracts in the cells (Figure 3). Results for concentrated extracts showed that exposure with NIVA-CYA 166 resulted in about 20% reduction of cell viability at the highest concentration, while the cell viability was little or not affected by exposure with the other six strains or the cyanobacterial growth medium (Figure 3).

### 2.2. Interference of Cytochrome P450-Mediated Metabolism of Estradiol Using Human Liver Microsomes (HLM)

**Figure 4:**
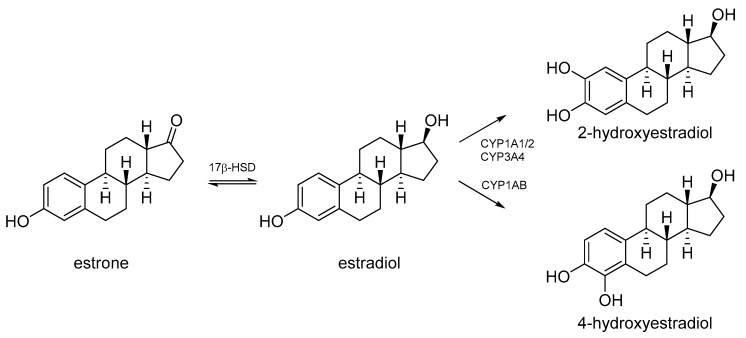
Main cytochrome P450-mediated pathways of estradiol to its 2- and 4-hydroxylated metabolites [45,46], as well as interconversion of estradiol and estrone.

**Figure 5:**
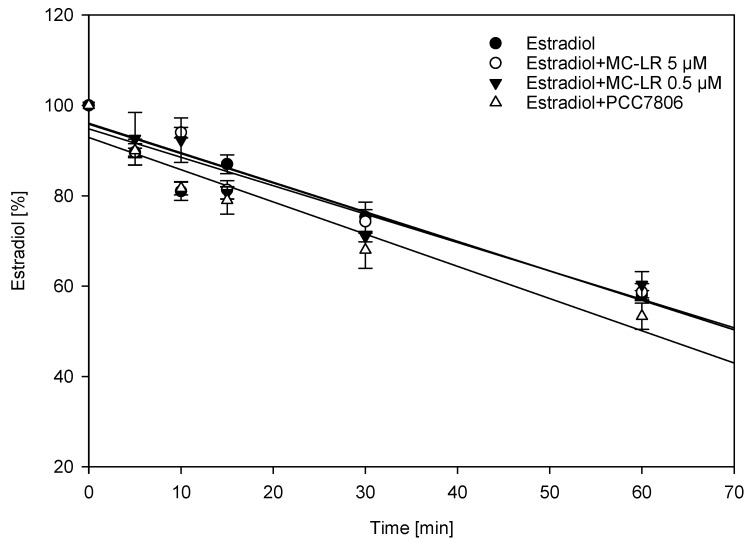
Estradiol depletion in human liver microsomes (HLM), either alone or in the presence of microcystin-LR (MC-LR) (0.5 µM and 5 µM) or *M. aeruginosa* PCC7806 extract. Data points are the mean ± SEM (n = 3) of three independent experiments.

We optimized a human liver microsomes (HLM) assay for the cytochrome P450-mediated biotransformation of estradiol to its main biotransformation product, 2-hydroxyestradiol, as well as 4-hydroxyestradiol (Figure 4) [43]. The levels of estrone and estriol were also monitored. Estrone is a natural weak estrogen which is in equilibrium with estradiol. Estrone can be a metabolic intermediate in the synthesis of estradiol. 17β-hydroxysteroid dehydrogenases (17β-HSD) catalyze interconversion of estradiol and estrone (Figure 4) [44]. The concentration of estradiol was chosen so it was depleted following first-order kinetics, and assay concentrations of its oxidation products as well as estradiol itself determined using a quantitative LC–MS/MS method (Figure 5). Interference of MCs and/or other *M. aeruginosa* metabolites with cytochrome P450-mediated biotransformation of estradiol was studied by co-incubation of estradiol with either pure MC-LR (0.5 or 5 µM) or an extract of *M. aeruginosa* PCC7806, which produces two main MC-variants, MC-LR and [D-Asp^3^] MC-LR [38]. The extract concentration of *M. aeruginosa* PCC7806 was adjusted so that the assay-concentration of MCs was about 0.5 µM.

Estradiol depletion was not significantly affected by the presence of either MC-LR or the PCC7806 extract at the tested concentrations. However, there was a reproducible trend to higher estradiol depletion when HLM were co-incubated with the PCC7806 extract for one hour (Figure 5).

The major estradiol oxidation products observed in the HLM assay were 2-hydroxyestradiol and estrone, while only traces of 4-hydroxyestradiol and estriol were detected (Figure S1). The concentrations of 2-hydroxyestradiol following co-incubation of estradiol with either 0.5 µM or 5 µM MC-LR, or *M. aeruginosa* PCC7806 extract, were significantly lower compared to when estradiol was incubated with HLM alone (Table 2, Figure S2, Table S5). In contrast, the concentrations of estrone were relatively higher when estradiol was co-incubated with either MC-LR or *M. aeruginosa* PCC7806 extract (Table 2). The relative increase in estrone was only statistically significant when estradiol was co-incubated with *M. aeruginosa* PCC7806 extract (Table 2). All background data from the experiments involving HLM are available in the Supplementary Material.

## 3. Discussion

Our screening of *Microcystis* and *Planktothrix* spp. culture extracts did not show substantial agonist or antagonist estrogen activity at the estrogen receptor level. Two strains showed statistically significant estrogen agonist activity when compared to Z8 medium. Furthermore, two strains showed a weak but statistically significant enhancement of the estradiol response, but we did not investigate further if this was due to synergism. None of these strains produced MCs, showing that the small but significant activity was not related to these toxins. We did not measure biomass, cell count, or the optical density of the cultures prior to extraction, but acknowledge that such measurements should have been performed. However, with respect to literature data investigating estrogen activity of MCs or *Microcystis* spp., we expected that extracts from visually dense cultures of the cyanobacteria would give a clear response in the estrogen-responsive RGAs provided that there are cyanobacterial metabolites or constituents that induce effects at the receptor level. Other limitations of the RGA-based screening approach were that the production of cyanobacterial metabolites might change over time depending on the growth stage, and metabolite production is also expected to be different in natural environments compared to laboratory conditions [38,47,48,49]. The reason for the observed agonist activity of the cyanobacterial culture medium Z8 is unclear as it does not contain any chemicals that are expected to possess estrogenic activities [42]. We believe that the origin of its weak biological activity could be due to contaminants from equipment used during the production of the medium, e.g., plastics, but did not investigate this further. However, it emphasizes that the activity of metabolites from the studied cyanobacterial strains on the estrogen receptor was minor. We did thus not aim to trace the relatively low activities in the estrogen responsive RGAs to specific cyanobacterial constituents.

While the study of estrogenic effects of cyanobacteria has been the subject of several reports, androgen- and glucocorticoid-type effects have not been studied in depth so far. However, the limited data did not support such effects from cyanobacterial extracts [19]. We only included androgen- and glucocorticoid-responsive RGAs in preliminary screenings that need further verification. However, the limited data indicated that the cyanobacterial extracts included in this study might induce androgen and glucocorticoid antagonist effects. The replicates variability in these initial screening was high and underlines the need for verification (Tables S1–S3).

MCs and extracts of *Microcystis* cultures reportedly reduce the reproduction in experimental fish [24,50,51]. The exact mechanism behind the effects on reproduction is not known and may involve effects on several endocrine pathways. Earlier work found that lyophilized *M. aeruginosa* but not pure MC-LR increased the expression of vitellogenin genes in zebrafish larvae [28], frequently interpreted as an indication of exposure to estrogenic compounds. Because of the lack of a clear estrogen activity at the receptor level, we investigated another mechanism that may lead to estrogenic effects, i.e., the modulation of hormone metabolism and biotransformation. Since we focused on effects of the cyanobacterial extracts on the estrogen receptor, it was natural to investigate the effect of MCs and a cyanobacterial extract on the microsomal biotransformation of estradiol. Since most toxicological studies have been carried out using the major MC-congener MC-LR, we also included this compound in our microsome trials, and co-incubated estradiol with 0.5 µM or 5 µM of the toxin. We also co-incubated estradiol with extract from *M. aeruginosa* strain PCC7806, and adjusted the extract concentration so that the total MCs assay concentration was about 0.5 µM. The PCC7806 strain has previously been shown to induce ED activity and produces MC-LR as principal MC congener. However, it is unknown if the effects were related to the MCs it produces, or other metabolites, but the data indicated that the ED activity was caused by metabolites other than MC-LR [28]. Although estradiol depletion was little affected by co-incubation with MC-LR or *M. aeruginosa* PCC7806, both the pure toxin and the cyanobacterial extract modulated the production of oxidized metabolites similarly (Table 2). Thus, the main product from microsomal cytochrome P450-mediated biotransformation, 2-hydroxyestradiol, was markedly reduced when the liver microsomes were co-incubated with either MC-LR or *M. aeruginosa* PCC7806. This effect was accompanied by an increase in relative estrone concentrations, and therefore overall estradiol depletion remained unaffected.

A large number of estradiol-metabolites have been shown, and they have different estrogenic potency [52,53]. The main hepatic phase-I metabolite 2-hydroxyestradiol has a considerably lower estrogenic potential than estradiol, estrone, 4-hydroxyestradiol, and many other tested estrogen metabolites [52]. Our findings indicate that MC-LR or extracts of a *Microcystis* culture may alter the metabolic transformation of estradiol to other metabolites with different estrogenic potential. It is therefore likely that a decreased formation of 2-hydroxyestradiol could lead to increased occurrence of estradiol or other metabolites with a higher estrogenic potency and hence induce an overall estrogenic effect. The degree of modulation of estradiol biotransformation was similar for co-incubation with 0.5 µM and 5 µM MC-LR (Table 2). However, at present we do not know the underlying mechanism(s) for the apparent modulation. While 2-hydroxylation is one of the principal microsomal biotransformation pathways for estradiol and catalyzed by CYP1A2 and CYP3A4, several other microsomal biotransformation products have been shown [46,54,55]. Future studies should therefore target as many biotransformation products of estradiol as possible, and should also investigate the possible interaction with relevant CYPs in detail. MC-LR and *Microcystis* extracts may also affect other biotransformation enzymes like sulphotransferase and steroid sulfatase. These enzymes are key enzymes in the regulation of the equilibrium between the free active form of estradiol and the much less active conjugates. MCs also affect the hypothalamic-pituitary-thyroid axis and the hypothalamic-pituitary-gonad axis in zebrafish [32,33,50]. Alterations in these pathways may interfere with normal homeostasis and hormone levels and hence explain the observed hormonal effects. Even the well-known inhibition of protein phosphatase PP2A [56,57,58,59] may affect the reproduction as the balance of phosphorylation/dephosphorylation of proteins are crucial for the regulation of intracellular signaling pathways and alterations may affect the development of gametocytes.

## 4. Conclusions

The interference of cyanobacterial metabolites from *Microcystis* and *Planktothrix* species with the estrogen receptor appears not to be a major biological effect. Future work should rather concentrate on studying interferences with the lesser-studied androgen and glucocorticoid receptors. Our data indicate that ED activity of MCs and/or other cyanobacterial compounds could arise from modulation of estrogen biotransformation as shown in HLM assays. Thus, MC-LR (and other MCs) may have ED activity.

## 5. Materials and Methods 

### 5.1. Chemicals and Reagents

Extraction of cultures: Methanol (MeOH) (gradient quality) was from Romil (Cambridge, UK).

RGAs: Cell culture reagents were supplied by Life Technologies (Paisley, UK). Standards used for RGAs (estradiol, testosterone, progesterone), phosphate buffered saline (PBS), MeOH, dimethyl sulfoxide (DMSO) and 3-(4,5-dimethylthiazol-2-yl)-2,5-diphenyltetrazolium bromide (MTT) were purchased from Sigma-Aldrich (Poole, Dorset, UK). Lysis reagents and luciferase assay system were obtained from Promega (Southampton, UK).

Estradiol biotransformation: NADP^+^, NADPH, D-glucose 6-phosphate sodium salt, D-glucose-6-phosphate dehydrogenase from baker’s yeast (Saccharomyces cerevisiae), HEPES buffer and ammonium formate were purchased from Sigma-Aldrich Norway (Merck Life Science AS, Oslo, Norway). Estradiol, 2-hydroxyestradiol, 4-hydroxyestradiol, estrone and estriol were purchased from Sigma-Aldrich (Millipore-Sigma; St. Louis, MO, USA). All stock solutions were prepared in MeOH at 1 mg/mL (estradiol, estrone, and estriol) or 0.5 mg/mL (2-hydroxyestradiol and 4-hydroxyestradiol). For estradiol, the working solution at 0.25 µg/mL was prepared by appropriate dilution from stock with MeOH. Stock solutions of 2-hydroxyestradiol, 4-hydroxyestradiol, estrone, and estriol were combined for the preparation of the mixed solution containing 2 µg/mL of each compound in 50% acetonitrile (MeCN). The mixture was used to prepare calibration standards in 50% MeCN at 12.5 ng/mL, 15 ng/mL, 75 ng/mL, 150 ng/mL, and 300 ng/mL for quantitative analysis (Table 3).

*ELISA:* Inorganic chemicals and organic solvents were reagent grade or better. MC-LR for the multihapten ELISA was from Enzo Life Sciences Inc. (Farmingdale, NY, USA). For further details refer to Samdal et al. (2014) [60]. 

### 5.2. Cultivation of Cyanobacterial Strains and Extraction

Twenty-five cyanobacterial strains (19 strains from *Microcystis* spp. and 6 strains from *Planktothrix* spp.) were purchased from The Norwegian Culture Collection of Algae, NORCCA, jointly maintained and owned by the Norwegian Institute for Water Research (NIVA) and the University of Oslo (Table S1). The *M. aeruginosa* PCC7806 strain was purchased from the Pasteur Institute (Paris, France) (Table S1). All strains were cultivated in Z8 medium [42] in 100 mL glass Erlenmeyer flasks in an incubator (IPP110plus, Memmert GmbH + Co.KG, Schwabach, Germany) at 18 °C with a 14/10 h light/dark photoperiod, using 1% of maximum light intensity.

For extract preparation, 3 mL of a cyanobacterial culture was transferred to a glass tube and stored at −20 °C overnight, then allowed to thaw at room temperature, and 3 mL of MeOH was added. The tube was then vortex-mixed for 20 s, sonicated for 5 min and centrifuged for 10 min at 1,000 × *g*. The supernatant was transferred to screw-cap liquid-chromatography (LC) glass vials and stored at −20 °C until use. For concentration of extracts, 1ml aliquots were dried under a gentle stream of nitrogen and re-dissolved in 50 µL of 50%MeOH in screw-cap LC glass vials and stored at −20 °C until use.

### 5.3. Microcystin ELISA

MCs production was assessed using an indirect competitive ELISA as described by Samdal et al. (2014) [60]. All samples and standard dilutions were done in duplicate and performed at ~20 °C. Absorbances were measured at 450 nm using a SpectraMax i3x plate reader (Molecular Devices, Sunnyvale, USA). For further details refer to Samdal et al. (2014) [60].

### 5.4. Cell Culture

Three reporter gene assay (RGA) cell lines were cultivated and used: the MMV-Luc (estrogen responsive), TARM-Luc (androgen responsive), and TGRM-Luc (glucocorticoid responsive). They were previously developed by transforming human mammary gland cell lines using the luciferase gene under the control of a steroid hormone inducible promoter, as reported by Willemsen et al. (2004) [61]. The cells were routinely grown in 75 cm^2^ tissue culture flasks (Nunc, Roskilde, Denmark), incubated at 37 °C in an atmosphere containing 5% CO_2_ and at 95% humidity. The MMV-Luc cell culture medium was made up of: Dulbecco’s Modified Eagle Medium (DMEM), supplemented with 10% fetal bovine serum (FBS) and 1% L-glutamine (2 mM). The TARM-Luc and TGRM-Luc cell culture medium was made up of DMEM Glutamax, supplemented with 10% FBS. The MMV-Luc culture medium was free from phenol red because of its weak estrogenic activity that may interfere with the assay.

### 5.5. Reporter-Gene Assays (RGAs)

Reporter gene assays (RGAs) are produced by transfecting cell lines with relevant receptors. A transactivation step with a signaling protein (the luciferase) is included. This step allows the measurement of receptor activation, distinguishing at the same time between agonist and antagonist behaviour [61]. The RGA procedures have previously been described by Frizzell et al. (2011) [62]. Briefly, cells were seeded at a concentration of 4 × 10^5^ cells/mL, 100 mL/well in white walled, clear, and flat bottomed 96-well plates (Greiner Bio-One, Fricken-hausen, Germany). RGA medium differed from that used for maintaining cell cultures in that hormone depleted serum was used instead of common FBS. After 24 h pre-incubation, cyanobacterial extract solutions as well as steroid hormone standards were added in triplicate to the cells at a final methanol concentration of 0.1%. Positive controls for the estrogen-, androgen-, and glucocorticoid-responsive RGAs were estradiol (1.36 ng/mL or 5 nM), testosterone (14.5 ng/mL) and cortisol (181 ng/mL), respectively. A solvent control (0.25% MeOH in deionized water, v/v) was also included. Antagonist tests included co-incubation of test compounds with the positive controls. The cells were incubated for 48 h. Supernatants were then discarded, and the cells were washed once with PBS. Then, 25 µL cell lysis buffer was added to each well. Reading was performed by adding 100 µL of luciferase substrate to each well and measuring luciferase activity with a Mithras Multimode Reader (Berthold, Other, Germany). Agonist response was measured relative to the solvent or cyanobacterial growth medium, and antagonist response was measured relative to the positive controls.

Estrogen-responsive RGAs and connected MTT cell viability assays were performed in triplicate and repeated in three independent exposures, while androgen-responsive and glucocorticoid-responsive RGAs and connected MTT cell viability assays were performed in triplicate, but in only one exposure each. 

### 5.6. MTT Cell Viability Assay

Flat-bottomed 96-well plates (Nunc) were seeded with 4×10^5^ cells/well of the appropriate RGA cell line. Cyanobacterial extracts were added to the cells after a pre-incubation period of 24 h at the desired concentration and maintaining a solvent concentration of 0.1% MeOH. Controls and extracts were incubated for 48 h. Supernatants were then discarded, and 50 µL of MTT solution (MTT stock solution/assay media ratio 1:6) added to each well and the cells incubated for another 3 h. Viable cells would convert the soluble and yellow dye MTT into insoluble and purple formazan. Supernatant were removed and 200 µL of DMSO added to each well to solubilize the formazan crystals. Optical density was measured at 570 nm (630 nm as reference) using a Sunrise spectrophotometer (TECAN, Switzerland). Cell viability was expressed as the relative absorbance of samples compared to the solvent control.

### 5.7. Human Liver Microsome (HLM) Incubation

In vitro metabolism trials were performed using commercially available human liver microsomes (HLM; No. X008067, Lot YAO; Celsis In Vitro Technologies, Baltimore, MD, USA). Estradiol (5 µM assay concentration) was incubated either alone or in presence of MC-LR solutions (0.5 µM and 5 µM assay concentration), as well as in presence of a *M. aeruginosa* PCC7806 extract. The extract was concentrated to obtain an in-assay concentration of MCs (sum of MC-LR and [D-Asp^3^]MC-LR) of approximately 0.5 µM. Individual incubations were carried out with 2 mg/mL of microsomal protein in a final volume of 1 mL containing a NADPH-generating system (0.91 mM NADPH, 0.83 mM NADP+, 19.4 mM glucose-6-phosphate, 1 U/mL glucose-6-phosphate dehydrogenase and 9 mM magnesium chloride hexahydrate) as well as an incubation buffer (45 mM Hepes, pH 7.4) at 37 °C for 60 min. Enzymatic reactions were stopped by adding aliquots from the individual incubations to ice-cold MeCN (1:1). All samples were vortex-mixed and placed on ice. They were centrifuged at 2,000 × *g* (Eppendorf, Hamburg, Germany) to precipitate proteins, filtered (0.22 µm Corning Costar Spin-X centrifuge tube filters, Corning Inc., NY, USA) and transferred to chromatography vials.

### 5.8. Quantification of Estradiol and Biotransformation Products Using LC–MS/MS

Estradiol and estradiol-related oxidative metabolites were determined by using an Agilent 1290 Infinity Binary UHPLC System with vacuum degasser and thermostatted column compartment (30 °C) coupled via an electrospray ionisation interface (ESI) to an Agilent 6470 TripleQ mass spectrometer (Agilent Technologies, Santa Clara, CA, USA). The instrument’s MassHunter Optimizer software (version B 8.0; Agilent) was used to evaluate the fragmentation patterns of the target compounds. The precursor-ion to product-ion transition, mass-to-charge ratio (*m/z*), fragmentor voltage (FV), collision energy (CE), and cell accelerator voltage (CAV) for multiple reaction monitoring (MRM) of all analytes (Table 3) were optimized by infusing a solution of the compounds isocratically with a flow rate of 0.4 mL/min (MeCN:water, 1/1, both containing 0.1% formic acid). Two transitions were monitored for each compound using dynamic multiple reaction monitoring (dMRM) according to Table 3.

The ES interface was operated in positive ion mode including a drying gas temperature of 300 °C, a drying gas flow of 10 L/min, a nebulizer gas pressure of 25 psi, a sheath gas temperature of 375 °C, a sheath gas flow of 12 L/min, a nozzle voltage of 1000 V, and a capillary voltage of 4 kV. A novel separation method was developed using a 150 × 2.1 mm i.d. Kinetex F5 (2.6 μm particles) HPLC column including a 0.5 μm KrudKatcher Ultra pre-column filter (Phenomenex). The target compounds were eluted using a mobile phase gradient consisting of water (A) and MeCN (B), both containing 0.1% formic acid. Chromatographic separation was achieved using isocratic elution at 2% B for 6 min followed by a linear gradient to 35% B over 2 min, and followed by a second linear gradient to 48% B over 10 min. After flushing the column for 3 min with 95% B, the mobile phase was returned to the initial conditions, and the column was eluted isocratically for an additional 2 min. The total run time was 24 min. The flow rate was 0.25 mL/min, the injection volume was 1 µL and the column temperature was maintained at 30 °C.

The biotransformation of estradiol to 2-hydroxyestradiol and estrone in the different groups (i.e., 0.5 µM MC-LR + 5 µM estradiol, 5 µM MC-LR + 5 µM estradiol or *M. aeruginosa* PCC7806 + 5 µM estradiol) was compared to the reference group (5 µM estradiol) using One-Way Analysis of Variance (ANOVA) followed by Dunnett’s test (JMP 14.0, SAS Institute Inc., Cary, NC, USA) (Figure S2, Table S4).

## Figures and Tables

**Table 1:** Cyanobacterial strains tested repeatedly in the estrogen-responsive reporter gene assays (RGAs) including information on production of microcystins (MCs).

Strain ID	Species	MCs ^a^
NIVA-CYA 431	*Microcystis novacekii*	No
NIVA-CYA 476	*Microcystis aeruginosa*	No
NIVA-CYA 22	*Microcystis aeruginosa*	No
PCC7806	*Microcystis aeruginosa*	Yes
NIVA-CYA 544	*Planktothrix prolifica*	Yes
NIVA-CYA 166	*Microcystis aeruginosa*	No
NIVA-CYA 31	*Microcystis aeruginosa*	Yes

^a^ based on literature data, confirmed by ELISA.

**Table 2:** Overall relative estrone and 2-hydroxyestradiol concentrations from co-incubation of estradiol with microcystin-LR (MC-LR) or *M. aeruginosa* PCC7806 extract with human liver microsomes (HLM) over 60 min. Numbers are the relative peak areas (in percentage, relative to incubation with estradiol alone) of the individual estradiol metabolites in incubations with or without MC-LR or *M. aeruginosa* PCC7806. Numbers are the grand mean of measurements at six time points and three independent experiments; the standard deviation is shown in parentheses.

Estradiol Metabolite	5 µM Estradiol (%)	5 µM Estradiol + 0.5 µM MC-LR (%)	5 µM Estradiol + 5 µM MC-LR	5 µM Estradiol + PCC7806
2-hydroxyestradiol	100	71 (±5) *	72 (±11) *	72 (±12) *
estrone	100	115 (±13)	105 (±10)	124 (±17) **

* significantly different from incubation with estradiol alone based on Dunnett’s test with *p* < 0.0001. ** significantly different from incubation with estradiol alone based on Dunnett’s test with *p* = 0.003.

**Table 3:** Instrument parameters for the LC–MS/MS analysis of estradiol and related metabolites.

Compound	Transition ^a^ [*m/z*]	CE ^b^	FV ^c^	CAV ^d^	RT ^e^
estriol	271.2 → 133.0 (157.0)	27	85	2	10.5
2-hydroxyestradiol	271.2 → 175.0 (149.0)	19	120	4	11.8
4-hydroxyestradiol	271.2 → 175.0 (149.0)	19	115	4	12.0
estradiol	255.2 → 159.0 (133.0)	19	100	2	13.5
estrone	271.2 → 133.0 (157.0)	31	70	4	15.1

^a^ precursor-ion to product-ion transitions, qualifier ion in parentheses. ^b^ collision energy in eV. ^c^ fragmentor voltage in V. ^d^ collision cell accelerator voltage in V. ^e^ retention time in min.

## Supplementary Materials

The following are available online at https://www.mdpi.com/2072-6651/12/4/228/s1, Table S1. Background information for cyanobacterial strains used in this study, including ID, species, origin and production of microcystins (MCs), Table S2. ER RGA summary, Table S3. AR RGA summary, Table S4. GR RGA summary, Table S5. *P*-values from post-hoc analyses (Dunnett’s test and Student’s *t* test) of the data set shown in Figure S2. Figure S1. Overlay of total ion chromatograms from LC–MS/MS analyses of estradiol incubations at 0 min and 60 min with human liver microsomes, Figure S2. One-way analysis of the relative production of 2-hydroxyestradiol and estrone in human liver microsomes, including Dunnett’s and Student’s *t* tests for post-hoc analysis.
