# Supplementary material for: Investigation of In Vitro Endocrine Activities of Microcystis and Planktothrix Cyanobacterial Strains
Source: Toxins (Basel). 2020 Apr 4;12(4):228. doi: 10.3390/toxins12040228 (PMC7232361; doi:10.3390/toxins12040228)
Supplement: Supplementary file 1 [file toxins-12-00228-s001.zip › toxins-744950 supplementary update/toxins-744950 supplementary proof done_VM and SU-1.docx]

Supplementary Materials: Investigation of *in Vitro* Endocrine Activities of *Microcystis* and *Planktothrix* Cyanobacterial Strains

**Vittoria Mallia, Lada Ivanova_,_ Gunnar S. Eriksen, Emma Harper, Lisa Connolly and Silvio Uhlig**

**Table S1.** Background information for cyanobacterial strains used in this study, including ID, species, origin and production of microcystins (MCs) according to ELISA.

| Strain ID | Species | Origin and year of isolation | MCs (ELISA) |
| --- | --- | --- | --- |
| NIVA-CYA 431* | *M. novacekii* | L. Victoria, Murchison Bay, Uganda, 2000 | no |
| NIVA-CYA 476* | *M. aeruginosa* | L. Victoria, Murchison Bay, Uganda, 2004 | no |
| NIVA-CYA 22* | *M. aeruginosa* | L. Mendota, Madison, WI, 1948 | no |
| PCC7806* | *M. aeruginosa* | Braakman Reservoirs, The Netherlands, 1972 | yes |
| NIVA-CYA 544* | *P. prolifica* | L. Steinsfjorden, Buskerud, Norway, 2004 | yes |
| NIVA-CYA 166* | *M. aeruginosa* | L. Hellesjøvatnet, Akershus, Norway, 1987 | no |
| NIVA-CYA 31* | *M. aeruginosa* | L. Little Rideau, Ontario, Canada, 1954 | yes |
| NIVA-CYA 24 | *P. prolifica* | L. Levrasjön, Kristianstad, Sweden, 1975 | no |
| NIVA-CYA 56/1 | *P. mougeotii* | L. Steinsfjorden, Buskerud, Norway, 1978 | no |
| NIVA-CYA 116 | *P. agardhii* | L. Årungen, Norway,1983 | no |
| NIVA-CYA 123/1 | *M. aeruginosa* | L. Mälaren, Sweden, 1983 | no |
| NIVA-CYA 140 | *M. aeruginosa* | Bendig's Pond, Bruno, Saskatchewan, Canada, 1975 | yes |
| NIVA-CYA 143 | *M. aruginosa* | L. Akersvatnet. Norway, 1984 | no |
| NIVA-CYA 144 | *M. cf. aeruginosa* | L. Borrevatnet, Vestfold, Norway, 1984 | no |
| NIVA-CYA 264 | *M. botrys* | L. Frøylandsvatnet, Rogaland, Norway, 1990 | yes |
| NIVA-CYA 279 | *M. cf. ichthyoblabe* | L. Østensjøvatnet, Oslo, Norway, 1990 | no |
| NIVA-CYA 432 | *Microcistys sp.* | L. Victoria, Murchison Bay, Uganda, 2000 | no |
| NIVA-CYA 475 | *M. aeruginosa* | L. Victoria, Murchison Bay, Uganda, 2000 | no |
| NIVA-CYA 478 | *M. aeruginosa* | L. Victoria, Murchison Bay, Uganda, 2000 | no |
| NIVA-CYA 482 | *M. aeruginosa* | L. Mburo, Uganda, 2004 | no |
| NIVA-CYA 598 | *P. prolifica* | L. Kolbotnvatnet, Akershus, Norway, 2007 | yes |
| NIVA-CYA 613 | *M. botrys* | L. Steinsfjorden, Buskerud, Norway, 2008 | yes |
| NIVA-CYA 632 | *P. rubescens* | L. Lyseren, Østfold, Norway, 2008 | yes |
| NIVA-CYA 754 | *M. aeruginosa* | Roodeplaat, South Africa, 2011 | no |
| NIVA-CYA 842 | *M. aeruginosa* | Hartbeespoort Dam, South Africa, 2013 | no |
| K-0540 | *M. aeruginosa* | Bagsværd Sø, Denmark, ? | no |

*selected strains, concentrated for further reporter gene assays.

**Table S2.** ER RGA summary.

| **ESTROGEN RESPONSIVE REPORTER GENE ASSAY** | | | | |
| --- | --- | --- | --- | --- |
| **Sample** | **Dilution** | **Agonism**  **% SC* response**  **(SEM)** | **Antagonism**  **% PC** response**  **(SEM)** | **Cell viability-Cytotoxicity**  **% response rel. to SC***  **(SEM)** |
| NIVA-CYA  598 | 1/400 | 8.4 (2.2) | 11.2 (3.0) | -10.3 (1.1) |
|  | 1/800 | 4.9 (1.6) | 8.3 (6.1) | -11.5 (3.2) |
| NIVA-CYA  842 | 1/400 | 3.3 (1.1) | 4.6 (1.9) | -16.6 (2.6) |
|  | 1/800 | 6.0 (3.0) | 3.4 (1.4) | -18.8 (6.5) |
| NIVA-CYA  613 | 1/400 | 1.9 (1.4) | 7.7 (3.3) | -15.0 (1.9) |
|  | 1/800 | 1.7 (0.5) | 8.3 (3.0) | -11.6 (0.5) |
| NIVA-CYA  431 | 1/400 | 15.7 (1.4) | 14.6 (2.8) | 1.8 (0.7) |
|  | 1/800 | 5.0 (1.1) | -11.2 (3.9) | 10.0 (3.7) |
| NIVA-CYA  140 | 1/400 | 8.5 (1.6) | 8.4 (1.9) | -6.1 (1.7) |
|  | 1/800 | 11.2 (6.4) | 3.1 (4.4) | -8.1 (0.7) |
| K-0540 | 1/400 | 0.8 (1.2) | 9.4 (3.7) | -3.8 (4.0) |
|  | 1/800 | 3.3 (1.8) | 8.5 (1.4) | -8.5 (2.3) |
| NIVA-CYA  475 | 1/400 | 2.1 (1.1) | 14.5 (2.0) | -13.2 (0.8) |
|  | 1/800 | -0.2 (0.8) | 11.8 (5.1) | -12.5 (3.5) |
| NIVA-CYA  476 | 1/400 | 1.3 (0.8) | 6.4 (2.7) | -0.4 (1.5) |
|  | 1/800 | 29.2 (2.8) | 6.4 (3.2) | 12.3 (5.8) |
| NIVA-CYA  22 | 1/800 | 20.5 (1.3) | 11.4 (3.6) | 2.7 (5.7) |
|  | 1/1600 | 14.1 (1.3) | 11.3 (5.4) | 11.6 (1.3) |
| NIVA-CYA  632 | 1/400 | 0.7 (0.9) | 24.0 (7.7) | 5.9 (2.8) |
|  | 1/800 | 0.1 (0.7) | -4.0 (17.3) | 7.3 (1.7) |
| NIVA-CYA  143 | 1/400 | 3.8 (1.6) | 18.3 (2.8) | -11.3 (2.5) |
|  | 1/800 | 8.7 (1.5) | 15.2 (11.0) | -10.1 (1.0) |
| NIVA-CYA  24 | 1/400 | 9.8 (0.6) | 5.8 (23.4) | -5.2 (1.5) |
|  | 1/800 | 0.4 (0.3) | -6.9 (11.1) | 4.1 (1.7) |
| PCC7806 | 1/400 | 12.6 (3.9) | -0.3 (22.5) | -11.2 (2.4) |
|  | 1/800 | 2.0 (0.7) | -7.3 (15.3) | 25.7 (3.3) |
| NIVA-CYA  478 | 1/400 | 9.3 (1.0) | 6.8 (0.5) | -6.6 (0.6) |
|  | 1/800 | -6.8 (1.3) | 15.7 (10.2) | -9.1 (1.3) |
| NIVA-CYA  544 | 1/400 | -2.2 (0.9) | 5.1 (1.6) | 10.6 (1.9) |
|  | 1/800 | 23.2 (11.9) | 10.7 (6.8) | 1.2 (3.2) |
| NIVA-CYA  279 | 1/400 | 6.9 (1.0) | -1.5 (4.6) | -6.2 (3.8) |
|  | 1/800 | -2.9 (0.8) | 11.5 (2.6) | -5.2 (2.9) |
| NIVA-CYA  144 | 1/400 | 5.2 (0.8) | 9.4 (4.3) | 1.9 (4.1) |
|  | 1/800 | 1.6 (0.6) | 9.8 (5.8) | 8.5 (1.6) |
| NIVA-CYA  482 | 1/400 | -1.6 (0.4) | 8.0 (5.6) | 3.4 (1.1) |
|  | 1/800 | -4.0 (0.2) | -6.2 (5.1) | 6.8 (2.2) |
| NIVA-CYA  754 | 1/400 | -6.6 (0.9) | 0.1 (9.6) | 1.9 (2.6) |
|  | 1/800 | -7.3 (0.6) | 3.1 (7.9) | 9.3 (1.5) |
| NIVA-CYA  166 | 1/400 | 22.3 (1.4) | -2.1 (5.7) | 14.4 (2.3) |
|  | 1/800 | -2.2 (2.0) | 4.2 (6.2) | 9.9 (0.4) |
| NIVA-CYA  31 | 1/400 | 32.5 (1.8) | 19.2 (8.6) | 20.1 (2.2) |
|  | 1/800 | 0.6 (0.5) | 7.6 (8.9) | 37.1 (8.5) |
| NIVA-CYA  432 | 1/400 | -2.4 (0.6) | -9.0 (2.7) | 18.6 (3.1) |
|  | 1/800 | -3.3 (1.0) | -4.1 (4.8) | 13.7 (2.5) |
| NIVA-CYA  56/1 | 1/400 | 11.7 (0.4) | 9.6 (1.1) | 10.6 (2.7) |
|  | 1/800 | -2.1 (1.3) | -1.5 (3.4) | 9.8 (1.5) |
| NIVA-CYA  116 | 1/400 | 0.9 (0.2) | -5.3 (7.2) | 2.1 (1.1) |
|  | 1/800 | -1.5 (1.3) | -2.5 (7.0) | 8.3 (2.4) |
| NIVA-CYA  264 | 1/400 | -4.2 (1.4) | 14.0 (9.0) | 10.1 (0.4) |
|  | 1/800 | -6.3 (1.0) | 10.0 (17.7) | 9.2 (1.7) |
| NIVA-CYA  123/1 | 1/400 | -1.0 (1.7) | 5.7 (26.5) | 3.4 (2.7) |
|  | 1/800 | -3.3 (0.6) | -11.8 (4.0) | 14.8 (5.2) |

*SC= solvent control (0.25% methanol in deionized water, v/v). **PC= positive control (Estradiol 1.36 ng/mL).

**Table S3.** AR RGA summary.

| **ANDROGEN RESPONSIVE REPORTER GENE ASSAY** | | | | |
| --- | --- | --- | --- | --- |
| **Sample** | **Dilution** | **Agonism**  **% SC* response**  **(SEM)** | **Antagonism**  **% PC** response**  **(SEM)** | **Cell viability-Cytotoxicity**  **% response rel. to SC***  **(SEM)** |
| NIVA-CYA  598 | 1/400 | 3.9 (1.3) | -61.4 (3.1) | -3.9 (3.8) |
|  | 1/800 | 4.0 (0.5) | -54.5 (4.3) | -1.4 (2.9) |
| NIVA-CYA  842 | 1/400 | 4.0 (1.2) | -46.1 (2.7) | -7.1 (3.3) |
|  | 1/800 | -0.2 (0.3) | -44.4 (8.1) | -11.0 (1.8) |
| NIVA-CYA  613 | 1/400 | 0.3 (0.6) | -34.6 (13.1) | -6.2 (0.8) |
|  | 1/800 | 3.1 (1.2) | -46.0 (2.7) | -3.0 (2.0) |
| NIVA-CYA  431 | 1/400 | 1.6 (1.1) | -54.1 (0.3) | -2.1 (3.9) |
|  | 1/800 | 5.1 (4.0) | -88.7 (1.6) | 1.8 (6.7) |
| NIVA-CYA  140 | 1/400 | 2.6 (0.8) | -78.4 (5.2) | 7.0 (1.5) |
|  | 1/800 | 3.3 (1.0) | -71.5 (6.2) | 4.4 (1.7) |
| K-0540 | 1/400 | 2.9 (0.8) | -55.4 (7.9) | 1.3 (2.1) |
|  | 1/800 | 3.6 (1.4) | -54.3 (4.4) | -4.6 (1.4) |
| NIVA-CYA  475 | 1/400 | 2.7 (1.9) | -47.6 (8.5) | -8.9 (3.2) |
|  | 1/800 | 1.2 (0.4) | -47.9 (6.9) | -13.2 (1.3) |
| NIVA-CYA  476 | 1/400 | 4.6 (1.7) | -44.0 (8.4) | -4.7 (2.1) |
|  | 1/800 | 3.0 (1.5) | -37.6 (1.9) | 0.8 (3.3) |
| NIVA-CYA  22 | 1/800 | 2.8 (1.1) | -33.4 (4.1) | -10.2 (3.6) |
|  | 1/1600 | 1.7 (1.0) | -24.4 (12.6) | 2.0 (0.9) |
| NIVA-CYA  632 | 1/400 | 3.2 (0.5) | -31.2 (5.8) | -4.3 (1.3) |
|  | 1/800 | 2.3 (0.5) | -25.5 (7.7) | -3.1 (0.6) |
| NIVA-CYA  143 | 1/400 | 3.1 (1.1) | -36.1 (6.6) | -3.5 (2.4) |
|  | 1/800 | 2.2 (0.7) | -21.3 (8.2) | -3.4 (1.2) |
| NIVA-CYA  24 | 1/400 | 3.4 (1.2) | -36.3 (4.4) | -4.3 (1.8) |
|  | 1/800 | 3.1 (1.5) | -39.9 (6.9) | 0.7 (3.8) |
| PCC7806 | 1/400 | 2.7 (1.1) | -50.9 (1.3) | 1.3 (1.5) |
|  | 1/800 | 1.2 (0.2) | -30.5 (1.0) | 4.0 (0.4) |
| NIVA-CYA  478 | 1/400 | 2.3 (0.3) | -34.6 (10.8) | -6.4 (0.8) |
|  | 1/800 | 2.1 (0.9) | -22.7 (1.1) | -6.3 (2.4) |
| NIVA-CYA  544 | 1/400 | 3.9 (0.6) | -17.4 (8.5) | -2.3 (3.2) |
|  | 1/800 | 3.7 (0.9) | -20.6 (6.2) | 42.3 (0.8) |
| NIVA-CYA  279 | 1/400 | 2.6 (0.7) | -8.7 (5.5) | 16.8 (17.6) |
|  | 1/800 | 4.1 (1.0) | -11.6 (2.9) | -1.9 (6.6) |
| NIVA-CYA  144 | 1/400 | 2.8 (0.9) | -25.9 (14.3) | -2.1(21.6) |
|  | 1/800 | 2.7 (0.5) | -34.6 (8.1) | -4.2 (1.8) |
| NIVA-CYA  482 | 1/400 | 1.4 (0.5) | 2.1 (2.3) | 2.8 (2.6) |
|  | 1/800 | 3.2 (0.8) | 11.3 (5.6) | -3.5 (2.7) |
| NIVA-CYA  754 | 1/400 | 3.5 (0.5) | 18.1 (14.7) | 1.8 (2.4) |
|  | 1/800 | 2.4 (0.5) | 9.9 (6.0) | -3.9 (4.5) |
| NIVA-CYA  166 | 1/400 | 2.8 (0.6) | 20.7 (5.3) | 0.8 (9.8) |
|  | 1/800 | 4.0 (0.9) | -1.3 (7.4) | -4.8 (2.1) |
| NIVA-CYA  31 | 1/400 | 3.1 (0.5) | 9.8 (14.3) | -5.7 (1.4) |
|  | 1/800 | 2.6 (0.4) | 22.7 (2.5) | 0.5 (4.0) |
| NIVA-CYA  432 | 1/400 | 1.7 (0.2) | 8.8 (14.5) | 7.2 (0.9) |
|  | 1/800 | 0.3 (0.4) | 6.1 (7.2) | -0.5 (3.1) |
| NIVA-CYA  56/1 | 1/400 | 5.2 (0.7) | 3.6 (3.8) | -4.7 (4.7) |
|  | 1/800 | 1.9 (0.4) | 1.6 (0.5) | 17.2 (11.3) |
| NIVA-CYA  116 | 1/400 | 2.3 (1.3) | 12.3 (7.8) | -9.4 (1.9) |
|  | 1/800 | 1.5 (0.6) | 4.6 (14.0) | -7.5 (1.3) |
| NIVA-CYA  264 | 1/400 | 2.6 (0.5) | 15.8 (13.2) | -4.5 (10.7) |
|  | 1/800 | 2.9 (0.7) | 5.0 (11.9) | -7.0 (3.6) |
| NIVA-CYA  123/1 | 1/400 | 2.5 (0.6) | 17.3 (18.2) | -5.3 (0.7) |
|  | 1/800 | 2.0 (1.4) | 28.3 (8.9) | 9.9 (7.8) |

*SC= solvent control (0.25% methanol in deionized water, v/v). **PC= positive control (Testosterone 14.5 ng/mL).

**Table S4.** GR RGA summary.

| **GLUCOCORTICOID RESPONSIVE REPORTER GENE ASSAY** | | | | |
| --- | --- | --- | --- | --- |
| **Sample** | **Dilution** | **Agonism**  **% SC* response**  **(SEM)** | **Antagonism**  **% PC** response**  **(SEM)** | **Cell viability-Cytotoxicity**  **% response rel. to SC***  **(SEM)** |
| NIVA-CYA  598 | 1/400 | 1.9 (0.7) | -7.6 (21.9) | 9.1 (9.3) |
|  | 1/800 | 0.4 (0.2) | 30.2 (27.4) | 4.2 (3.9) |
| NIVA-CYA  842 | 1/400 | 0.1 (0.0) | -10.6 (33.3) | 7.9 (7.4) |
|  | 1/800 | 0.0 (0.0) | 17.1 (34.0) | 13.0 (7.9) |
| NIVA-CYA  613 | 1/400 | 0.0(0.0) | 14.0 (13.5) | 3.7 (6.1) |
|  | 1/800 | -0.1 (0.0) | -39.4 (31.2) | -4.8 (2.3) |
| NIVA-CYA  431 | 1/400 | 0.0 (0.0) | -17.1 (16.6) | -2.8 (2.5) |
|  | 1/800 | 0.2 (0.2) | -12.3 (21.3) | -7.0 (7.5) |
| NIVA-CYA  140 | 1/400 | 0.3 (0.2) | -5.7 (26.6) | 17.2 (6.6) |
|  | 1/800 | 1.7 (1.6) | 31.6 (37.3) | 7.7 (0.7) |
| K-0540 | 1/400 | 0.2 (0.1) | -52.4 (9.9) | 7.6 (5.4) |
|  | 1/800 | 0.0 (0.0) | -23.4 (16.9) | 4.1 (15.7) |
| NIVA-CYA  475 | 1/400 | 0.0 (0.0) | -20.6 (4.8) | 20.4 (20.5) |
|  | 1/800 | 2.6 (1.4) | -20.0 (5.1) | 8.7 (8.2) |
| NIVA-CYA  476 | 1/400 | 0.1 (0.0) | -28.0 (11.9) | 8.2 (6.0) |
|  | 1/800 | 0.0 (0.0) | 32.4 (25.1) | -4.4 (6.8) |
| NIVA-CYA  22 | 1/800 | 0.0 (0.0) | -19.3 (8.1) | -9.2 (4.5) |
|  | 1/1600 | 0.0 (0.0) | 9.9 (54.5) | 4.5 (1.4) |
| NIVA-CYA  632 | 1/400 | 0.0 (0.0) | -39.9 (11.0) | -9.4 (6.2) |
|  | 1/800 | 0.0 (0.0) | -25.4 (28.8) | -14.8 (12.2) |
| NIVA-CYA  143 | 1/400 | 0.1 (0.1) | -88.6 (8.2) | -3.7 (8.6) |
|  | 1/800 | 0.1 (0.1) | -29.9 (17.5) | -1.0 (7.1) |
| NIVA-CYA  24 | 1/400 | 0.2 (0.2) | 17.8 (9.2) | -6.5 (3.9) |
|  | 1/800 | 0.1 (0.1) | -15.2 (30.4) | -10.1 (8.6) |
| PCC7806 | 1/400 | 0.0 (0.0) | 0.7 (19.6) | -15.0 (5.8) |
|  | 1/800 | 0.0 (0.0) | -40.0 (18.5) | 7.5 (19.0) |
| NIVA-CYA  478 | 1/400 | 0.7 (0.6) | -7.9 (26.5) | -4.6 (12.7) |
|  | 1/800 | 0.2 (0.1) | -11.3 (37.3) | -7.7 (3.1) |
| NIVA-CYA  544 | 1/400 | 0.1 (0.0) | **7.7 (20.5)** | 4.2 (13.1) |
|  | 1/800 | 0.0 (0.0) | -76.7 (13.0) | 7.0 (10.3) |
| NIVA-CYA  279 | 1/400 | 0.0 (0.0) | -54.0(19.3) | -1.3 (16.2) |
|  | 1/800 | 0.0 (0.0) | 25.8(14.2) | -2.0 (6.1) |
| NIVA-CYA  144 | 1/400 | 0.0 (0.0) | -79.1 (9.0) | -16.1 (4.8) |
|  | 1/800 | 0.1 (0.0) | 26.7 (14.9) | -18.9 (7.3) |
| NIVA-CYA  482 | 1/400 | 0.1 (0.1) | 32.2 (19.8) | -9.2 (5.5) |
|  | 1/800 | 0.1 (0.1) | -57.3 (8.5) | -0.4 (14.0 |
| NIVA-CYA  754 | 1/400 | 0.0 (0.0) | -43.3 (15.8) | -5.9 (5.2) |
|  | 1/800 | 0.0 (0.0) | -13.0 (10.0) | -3.5 (4.2) |
| NIVA-CYA  166 | 1/400 | 0.0 (0.0) | -23.1 (24.6) | -9.9 (4.3) |
|  | 1/800 | 0.6 (0.1) | 28.0 (22.3) | -5.5 (1.3) |
| NIVA-CYA  31 | 1/400 | 9.1 (2.0) | -31.6 (32.1) | -12.6 (1.2) |
|  | 1/800 | 0.0 (0.0) | -55.9 (32.0) | -4.2 (4.7) |
| NIVA-CYA  432 | 1/400 | 0.1 (0.1) | -74.1 (19.0) | -5.0 (4.6) |
|  | 1/800 | 0.0 (0.0) | -38.9 (18.5) | -2.6 (3.8) |
| NIVA-CYA  56/1 | 1/400 | -0.1 (0.0) | -26.5 (11.5) | -18.9(1.8) |
|  | 1/800 | 0.0 (0.0) | -2.5 (25.2) | -10.2 (3.4) |
| NIVA-CYA  116 | 1/400 | 0.2 (0.1) | -40.6 (28.5) | -16.2 (3.0) |
|  | 1/800 | 0.3 (0.2) | -20.6 (11.0) | -10.4 (2.4) |
| NIVA-CYA  264 | 1/400 | 0.2 (0.1) | -3.0 (9.5) | -12.1 (4.3) |
|  | 1/800 | 0.1 (0.0) | -69.8 (17.1) | -5.4 (3.9) |
| NIVA-CYA  123/1 | 1/400 | 0.1 (0.0) | 17.7 (30.1) | -17.8 (3.4) |
|  | 1/800 | 0.1 (0.0) | 39.3 (5.2) | 0.2 (11.4) |

*SC= solvent control (0.25% methanol in deionized water, v/v). **PC= positive control (Cortisol 181 ng/mL).

**Figure S1.** Overlay of total ion chromatograms from LC–MS/MS analyses of estradiol incubations at 0 min (red line) and 60 min (black line) with human liver microsomes (HLM). The concentration of individual compounds in the calibration standard was 125 ng/ml (green line).

2-hydroxyestradiol

estrone

**Figure S2.** One-way analysis of the relative production of 2-hydroxy-estradiol and estradione in human liver microsomes, including Dunnett’s and Student’s t tests for post-hoc analysis. The data set consists of the following groups: **1**, 5 µM estradiol only (reference); **2**, 5 µM estradiol + 5 µM MC-LR; **3**, 5 µM estradiol + 0.5 µM MC-LR; **4**, 5 µM estradiol + *M. aeruginosa* PCC7806 containing 5 µM MC-LR.

**Table S5.** *P*-values from post-hoc analyses (Dunnett’s test and Student’s *t* test) of the data set shown in Figure S2 using estradiol as reference (n=18, i.e. three replicate experiments including six time points). *P*-values that identify statistically significant differences relative to incubations with estradiol alone are shown in bold.

| **Estradiol Metabolite** | **5 µM estradiol +**  **0.5 µM MC-LR** | | **5 µM estradiol +**  **5 µM MC-LR** | | **5 µM estradiol +**  **PCC7806** | |
| --- | --- | --- | --- | --- | --- | --- |
|  | ***P*-value (Dunnett’s)** | ***P*-value (Student’s *t*)** | ***P*-value**  **(Dunnett’s)** | ***P*-value (Student’s *t*)** | ***P*-value**  **(Dunnett’s)** | ***P*-value (Student’s *t*)** |
| 2-hydroxyestradiol | <0.0001 | <0.0001 | <0.0001 | <0.0001 | <0.0001 | <0.0001 |
| estrone | 0.096 | 0.038 | 0.845 | 0.508 | 0.003 | 0.001 |
